# Risk analysis of poor prognosis and follow-up observation of children with *Mycoplasma pneumoniae* pneumonia complicated by plastic bronchitis

**DOI:** 10.3389/fped.2025.1565773

**Published:** 2025-06-27

**Authors:** Chunlin Zheng, Zhidong Zheng, Xiangrong Hu, Yizhu Huang, Yimin Han

**Affiliations:** Department of Pediatrics, The First Hospital of Putian City, Putian, China

**Keywords:** *mycoplasma pneumoniae* pneumonia, plastic bronchitis, poor prognosis, risk factors, follow-up, pulmonary function, D-dimer

## Abstract

**Background:**

Studies on the risk factors for poor prognosis in pediatric plastic bronchitis (PB) and medium- to long-term follow-up are relatively limited. The aim of this study was to investigate the risk factors for poor prognosis of *Mycoplasma pneumoniae* pneumonia (MPP) complicated by PB prognosis in children and conduct a detailed follow-up study.

**Methods:**

This was a retrospective study of children diagnosed with MPP complicated by PB at the First Hospital of Putian City from January 2022 to June 2024. Clinical data during hospitalization and after discharge were collected. Patients were assigned to the good prognosis group if both pulmonary imaging and pulmonary function tests one month after discharge were normal or the poor prognosis group if one or both tests were abnormal. Clinical manifestations and laboratory findings were compared between the two groups.

**Results:**

This study included 62 pediatric patients, with a median age of 8.1 years, of which 35 were male. The poor prognosis group comprised 40 patients (64.5%), and male children accounted for 57.5% (23/40) of this group. Logistic regression analysis indicated that longer fever duration (OR = 2.871) and higher D-dimer level (OR = 1.770) were independent risk factors for poor prognosis in children with PB. ROC curve analysis further revealed that a fever duration of 10.55 days (AUC = 0.852) and a D–dimer level of 5.61 µg/ml (AUC = 0.806) provided optimal prediction of poor prognosis.All patients presented with abnormal pulmonary imaging findings at admission, and 43 (69.4%) had abnormal pulmonary function on the day of discharge; follow-up revealed that 33 patients (53.2%) had abnormal pulmonary imaging findings, and 28 patients (45.2%) had abnormal pulmonary function one month after discharge. Six months after discharge, 13 patients (21.0%) continued to exhibit either pulmonary lesions or impaired pulmonary function.

**Conclusion:**

A fever duration longer than 10.55 days and a D-dimer level higher than 5.61 µg/ml were independent risk factors for poor prognosis in children with MPP complicated by PB. Children with PB caused by *Mycoplasma pneumoniae* may experience long-term sequelae, necessitating close follow-up and personalized rehabilitation treatment.

## 1 Introduction

With the increasing popularity of pediatric bronchoscopy technology, there has been a corresponding increase in the diagnoses of plastic bronchitis (PB) in children (1–3). PB, a rare respiratory disease, has diverse clinical manifestations, and affected children face significant therapeutic challenges, markedly impacting their health and quality of life. *Mycoplasma pneumoniae* (MP) is a common pathogenic cause of respiratory infection. At present, the focus of research on PB caused by MP has been mainly on the interpretation of clinical manifestations and the exploration of risk factors for PB (4, 5). The focus of a small number of studies has been on the medium- to long-term prognosis of PB, but these studies have either involved a small number of patients or relied simply on pulmonary imaging to judge the prognosis (6–8). Therefore, there is a lack of in-depth research on the follow-up prognosis and evaluation and prediction of risk factors for PB. Here, we aimed to conduct a rigorous retrospective analysis and, on the basis of the fact that all the enrolled children had *Mycoplasma pneumoniae* pneumonia (MPP) complicated by PB, the pulmonary imaging and pulmonary function of the children 1 month after discharge were used as markers to judge the prognosis of PB and further explore the risk factors affecting the poor prognosis of MPP complicated by PB in children to aid in treatment decision-making. Additionally, through six-month longitudinal observations, this study more comprehensively revealed the natural disease progression and prognostic patterns of PB. This study provides a more scientific basis for the formulation of precision clinical treatment and long-term management strategies for PB, thereby further improving the treatment outcomes and quality of life of children with PB.

## 2 Methods

### 2.1 Study subjects

This was a retrospective study of pediatric patients diagnosed with MPP complicated by PB at the Department of Pediatrics, The First Hospital of Putian City, from January 2022 to June 2024. The patients were divided into poor prognosis and good prognosis groups on the basis of abnormal pulmonary imaging findings and pulmonary function test results one month after discharge. Specifically, abnormal findings were categorized on the basis of internationally recognized standards, including bronchopneumonia, atelectasis, and pulmonary necrosis, as indicated by chest CT scans, as well as abnormal results of key indicators in pulmonary function tests, such as forced expiratory volume in the first second (FEV1), forced vital capacity (FVC), and the FEV1/FVC ratio. Patients with abnormalities in either pulmonary imaging or pulmonary function were classified into the poor prognosis group, whereas those with normal findings in both aspects were classified into the good prognosis group.

The inclusion criteria were as follows: (1) fever and/or cough, pulmonary imaging findings suggestive of pneumonia, and pathogen detection indicating that MP is the sole pathogen meeting the diagnostic criteria for MPP (9); (2) extraction of endobronchial casts resembling the bronchial tree under electronic bronchoscopy meeting the criterion for PB (10); and (3) age ranging from 5 to 14 years. The exclusion criteria were as follows: (1) no MP infection or mixed infections as indicated by etiological test results; (2) a history of recurrent wheezing, pulmonary insufficiency, congenital heart disease or operation after congenital heart disease; (3) aspiration of exogenous foreign bodies; (4) new pulmonary lesions unrelated to the primary disease identified during follow-up; and (5) missing or incomplete data.This study was approved by the Medical Ethics Committee of The First Hospital of Putian City (Approval Number: 2024-090).

### 2.2 Detection method

#### 2.2.1 Bronchoscopy examination and treatment

In accordance with the Chinese Pediatric Flexible Bronchoscopy Procedure Guidelines, patients who were eligible for bronchoscopic intervention and without any contraindications were included (11). Flexible electronic bronchoscopy (QG-3320 and QG-3430, Zhuhai Shixin) was performed under general anesthesia with a laryngeal mask airway (PLMA2010SDB and PLMA2512SDB, Kangyuan Laryngeal Mask Airway) to extract mucous plugs via negative pressure aspiration or foreign body forceps (FG-12E-10EE4, Changmei Medtech), followed by alveolar lavage.

#### 2.2.2 Etiological detection

Alveolar lavage fluid (5 ml) was collected for targeted next-generation sequencing (tNGS) (KM MiniSeqDx-CN, Kingcreate Biotech) detection and sputum culture examination. The pathogens detected by tNGS included bacteria, viruses, fungi, and special pathogens such as MP, as well as the detection of MP resistance-related genes. Sputum culture involved bacterial culture, which was plated on blood agar and incubated for 16–18 h, followed by identification of isolated single colonies via the Diels Smart ms 5,020 mass spectrometry method.

#### 2.2.3 Pulmonary function testing

Patients with absolute contraindications to pulmonary function tests, such as pneumothorax and bullae, were excluded, and the German Jaeger MasterScreen pulmonary function instrument was used to perform maximal expiratory flow‒volume (MEFV) curve pulmonary function tests. The study subjects were of Asian descent. According to the pediatric pulmonary function series guidelines (II) developed by the Pulmonary Function Collaborative Group of the Respiratory Group of the Pediatric Branch of the Chinese Medical Association in 2016 (12), pulmonary ventilation function parameters were divided into two parts: (1) The maximal vital capacity (VCMAX), forced vital capacity (FVC), and forced expiratory volume in the first second (FEV1) values ≥80% of the predicted value were considered normal; values of 60%–79% of the predicted value were considered to indicate a mild decrease; values of 40%–59% of the predicted value were considered to indicate a moderate decrease; and values <40% of the predicted value were considered to indicate a severe decrease, mainly reflecting obstructive, restrictive, or mixed ventilation dysfunction. The diagnosis of obstructive lesions was based on the premise that the actual FEV1/FVC value was less than 92% of the predicted value and that the degree of damage was measured as the actual FEV1 value/predicted value. (2) When the above main parameters were normal, if the values of two of the following three indicators were abnormal, small airway dysfunction was considered: forced expiratory flow at 50% of the FVC exhaled (FEF50), forced expiratory flow at 75% of the FVC exhaled (FEF75), and maximal midexpiratory flow (MMEF). The specific classification was as follows: values ≥65% of the predicted value were considered normal; values of 55%–64% of the predicted value were considered to indicate a mild decrease; values of 45%–54% of the predicted value were considered to indicate a moderate decrease; and values <45% of the predicted value were considered to indicate a severe decrease.

#### 2.2.4 Pulmonary imaging and inflammatory blood marker detection

Pulmonary imaging was performed by computed tomography (Philips Brilliance CT). Laboratory tests were conducted as follows: 5 ml of fasting venous blood was collected from the child, and the white blood cell (WBC) count was determined on the basis of the principle of electrical impedance. C-reactive protein (CRP) and D-dimer (D-D) levels [with a normal range for (D-D) levels: 0–0.55 µg/ml] were measured with the immunoturbidimetric method, and lactate dehydrogenase (LDH) was assessed with the lactate method.

### 2.3 Treatment methods

After admission, all patients received standardized treatment, including oral azithromycin at a dose of 10 mg/kg per administration, once a day for 3 consecutive days, followed by a 4-day pause, resulting in one course of treatment, for a total of 2–3 courses; on the basis of the results of pathogen detection, for patients with macrolide resistance, azithromycin was discontinued after informed consent was obtained from the patients' parents, and doxycycline was used instead at a dose of 2 mg/kg per administration, twice a day for a full course of 10 days; this is in line with the recommendations for the rational treatment of MPP in children according to Tsai et al. (13). According to the expert consensus on the use of glucocorticoid nebulized inhalation therapy in pediatrics (revised in 2018) (14), for children in the recovery period of MPP who still exhibit airway hyperresponsiveness or localized atelectasis, nebulized inhalation of budesonide suspension at a dosage of 0.5–1.0 mg/day can be continued for 1–3 months postdischarge.

### 2.4 Observation indicators

Clinical data were collected for the two groups of children during hospitalization, at one month after discharge, and at six months after discharge. The data included pulmonary imaging (CT) within 24 h of admission, pulmonary function data (VCMAX, FVC, FEV1, FEF50, FEF75, and MMEF) on the day of discharge, and both pulmonary imaging (CT) and pulmonary function one month and six months postdischarge; laboratory test indicators within 24 h of admission included WBC count, CRP level, LDH level, and D-D level; and data on other characteristics including sex, age, fever duration (the length of time with fever), peak temperature (the highest temperature during fever), cough, wheezing, dyspnea, chest pain, three-sign (retraction of the intercostal spaces during inspiration), decreased breath sounds, types of extrapulmonary complications, oxygen therapy (oxygen administration via nasal cannula or mask), macrolide drug resistance (resistance and type of resistance gene), and the number of bronchoscopic interventions (single or multiple).

### 2.5 Statistical analysis

SPSS 22.0 software was used for statistical analysis. Enumeration data are presented as the number of cases and percentage (%), with the chi-square test or Fisher's exact test used to compare differences between groups. Normally distributed data are expressed as the means ± standard deviations (SDs), and the independent samples *t* test was used for intergroup comparisons. Nonnormally distributed data are expressed as medians (P25, P75), and the nonparametric Mann–Whitney *U* test was used for intergroup comparisons. Logistic regression analysis was performed to identify influencing factors, and the predictive value of risk factors was analyzed via receiver operating characteristic (ROC) curves, which were plotted with GraphPad Prism. All tests were two-sided, and a *p* value of less than 0.05 was considered statistically significant.

## 3 Results

### 3.1 Clinical manifestations

In total, 69 pediatric patients diagnosed with MPP complicated by PB at the Department of Pediatrics, The First Hospital of Putian City, from January 2022 to June 2024 were identified. After screening, 7 children were excluded from the study. Five children were excluded due to mixed pathogen infections, 1 was excluded due to comorbid asthma, and 1 child was excluded due to incomplete data owing to noncompliance with follow-up. A total of 62 children were ultimately included in the study, with 40 in the poor prognosis group and 22 in the good prognosis group.

Among the 62 children with PB, 40 were male and 22 were female, with ages ranging from 5.1 to 13 years (median, 8.1 years). Male children accounted for 54.5% (12/22) of the good prognosis group and 57.5% (23/40) of the poor prognosis group. There was no significant difference in sex between the two groups. The median ages of the children in the good and poor prognosis groups were 9.2 years and 7.8 years, respectively. The proportions of patients with poor prognosis were 75.9%, 56.3%, and 52.9% in the 5–7.9 years, 8–9.9 years, and 10–13 years age groups, respectively. There was no significant difference in the distribution of age groups between the two groups.

In this study, children with MPP complicated by PB mainly presented with fever (100.0%) and cough (91.9%). The duration of fever in the poor prognosis group was significantly longer than that in the good prognosis group (11.82 ± 1.81 vs. 9.63 ± 0.98, *p* < 0.001), but there was no difference in the peak temperature between the two groups. Decreased breath sounds (38.7%) and dyspnea (27.4%) were also observed in some children. Other clinical manifestations included wheezing (19.4%), the three depressions sign (17.7%), and chest pain (6.5%). Additionally, there were no significant differences in cough, decreased breath sounds, dyspnea, wheezing, the three depression sign or chest pain between the two groups (*p* > 0.05). The extrapulmonary complications involved multiple systems, with thrombocytopenia in 6 patients (9.68%) and erythema multiforme in 18 patients (29.0%). The incidence of immune thrombocytopenia in the poor prognosis group was 12.5% (5/40), and the incidence of erythema multiforme was 32.5% (13/40). There was no significant difference in extrapulmonary complications between the two groups (*p* > 0.05) (Table 1).

**Table 1:** Comparison of clinical characteristics and treatments of the two groups.

Project	Total (*n* = 62)	Poor prognosis group (*n* = 40)	Good prognosis group (*n* = 22)	*χ*^2^/T/Z	*P*
General information					
Sex (m/f, *n*)				0.050	0.822
Male (*n*, %)	35 (56.5%)	23 (57.5%)	12 (54.5%)		
Female (*n*, %)	27 (43.5%)	17 (42.5%)	10 (45.5%)		
Age (years)	8.1 (6.6, 10.2)	7.8 (6.5, 9.7)	9.2 (6.4, 10.4)	−1.030	0.303
Age Group (*n*, %)				3.103	0.212
5–7.9 (years)	29 (46.8%)	22 (55.0%)	7 (31.8%)		
8–9.9 (years)	16 (25.8%)	9 (22.5%)	7 (31.8%)		
10–13 (years)	17 (27.4%)	9 (22.5%)	8 (36.4%)		
Clinical manifestations					
Fever (*n*, %)	62 (100.0%)	40 (100.0%)	22 (100.0%)	/	/
Fever duration (days)	11.05 ± 1.88	11.82 ± 1.81	9.63 ± 0.98	6.191	<0.001
Peak temperature (°C)	39.7 ± 0.6	39.8 ± 0.6	39.6 ± 0.6	1.420	0.161
Cough	57 (91.9%)	36 (90.0%)	21 (95.5%)	0.570	0.789
Wheezing	12 (19.4%)	9 (22.5%)	3 (13.6%)	0.714	0.611
Dyspnea	17 (27.4%)	14 (35.0%)	3 (13.6%)	3.255	0.071
Chest pain	4 (6.5%)	2 (5.0%)	2 (9.1%)	0.394	0.931
Three depressions sign	11 (17.7%)	10 (25.0%)	1 (4.5%)	4.069	0.095
Decreased breath sounds	24 (38.7%)	18 (45.0%)	6 (27.3%)	1.880	0.170
Extrapulmonary complications					
Thrombocytopenia (*n*, %)	6 (9.7%)	5 (12.5%)	1 (4.5%)	1.027	0.572
Erythema multiforme (*n*, %)	18 (29.0%)	13 (32.5%)	5 (22.7%)	0.658	0.417
Laboratory test results					
WBC (×10^9^/L)	8.45 ± 2.63	8.50 ± 2.77	8.34 ± 2.40	0.225	0.823
CRP (mg/L)	41.78 ± 11.65	44.11 ± 12.03	37.53 ± 9.80	2.190	0.032
LDH (U/L)	480.60 ± 57.75	494.00 ± 45.02	456.23 ± 70.42	2.273	0.030
D-D (mg/ml)	6.32 (4.29, 7.89)	6.96 (5.89, 8.25)	4.11 (3.31, 6.44)	−3.958	<0.001
Clinical interventions					
Oxygen therapy (*n*, %)	20 (32.3%)	17 (42.5%)	3 (13.6%)	5.411	0.020
Macrolide resistance (*n*, %)	50 (80.6%)	36 (90.0%)	14 (63.6%)	6.320	0.029
Multiple Bronchoscopic Interventions (*N*, %)	41 (66.1%)	31 (77.5%)	10 (45.5%)	6.507	0.011

Enumeration data are expressed as numbers (percentages). Measurement data are expressed as the median (P25, P75) or mean ± SD.

WBC, white blood cell; CRP, C-reactive protein; LDH, lactate dehydrogenase; D-D, D-dimer.

### 3.2 Laboratory tests

Significant differences in the levels of C-reactive protein (CRP), lactate dehydrogenase (LDH), and D-dimer (D-D) were observed between the poor prognosis group and the good prognosis group of children with PB (*p* < 0.05). The levels of CRP (44.11 ± 12.03 vs. 37.53 ± 9.80 mg/L, *p* = 0.032), LDH (494.00 ± 45.02 vs. 456.23 ± 70.42 U/L, *p* = 0.030), and D-D [6.96 [5.89, 8.25] vs. 4.11 [3.31, 6.44] µg/ml, *p* < 0.001] were significantly higher in the poor prognosis group than in the good prognosis group. There was no significant difference in the white blood cell (WBC) counts of the two groups (Table 1).

### 3.3 Treatments

Among the 62 patients, macrolide resistance was observed in 50 patients (80.6%), with the resistance gene family being 23S rRNA:A2063G; 20 patients (32.3%) required oxygen therapy, and 41 patients (66.1%) required multiple bronchoscopic interventions. The proportions of patients in the poor prognosis group who required oxygen therapy [17 [42.5%] vs. 3 [13.6%], *p* = 0.020], had macrolide resistance [36 [90.0%] vs. 14 [63.6%], *p* = 0.029], and had multiple bronchoscopic interventions [31 [77.5%] vs. 10 [45.5%], *p* = 0.011] were significantly higher than those in the good prognosis group (Table 1).

### 3.4 Multivariate logistic regression analysis of the two groups of children

We included indicators that were significantly different between the poor and good prognosis groups as independent variables, the presence of abnormalities on pulmonary imaging or pulmonary function one month after discharge (i.e., poor prognosis group and good prognosis group) as the dependent variable in the multivariate logistic regression analysis. The results indicated that a longer fever duration (OR = 2.871, 95% CI: 1.327–6.212, *p* < 0.05) and higher D-D levels (OR = 1.770, 95% CI: 1.050–2.982, *p* < 0.05) were independent risk factors for children with PB who had not recovered one month after discharge (Table 2).

**Table 2:** Multivariate logistic regression analysis of risk factors for poor prognosis.

Factors	B	SE	Wald	*P*	OR (95% CI)
Fever duration	1.055	0.394	7.174	0.007	2.871 (1.327–6.212)
Respiratory support	1.336	1.005	1.765	0.184	3.803 (0.530–27.289)
Macrolide resistance	0.252	1.071	0.055	0.814	1.286 (0.158–10.492)
Multiple bronchoscopic interventions	−1.215	0.949	1.642	0.200	0.297 (0.046–1.903)
CRP level	0.016	0.048	0.113	0.737	1.016 (0.924–1.118)
LDH level	0.005	0.010	0.313	0.576	1.005 (0.986–1.025)
D-D level	0.571	0.266	4.599	0.032	1.770 (1.050–2.982)
Constant	−17.024	5.815	8.569	0.003	

### 3.5 ROC curve analysis of risk factors

**Figure 1:**
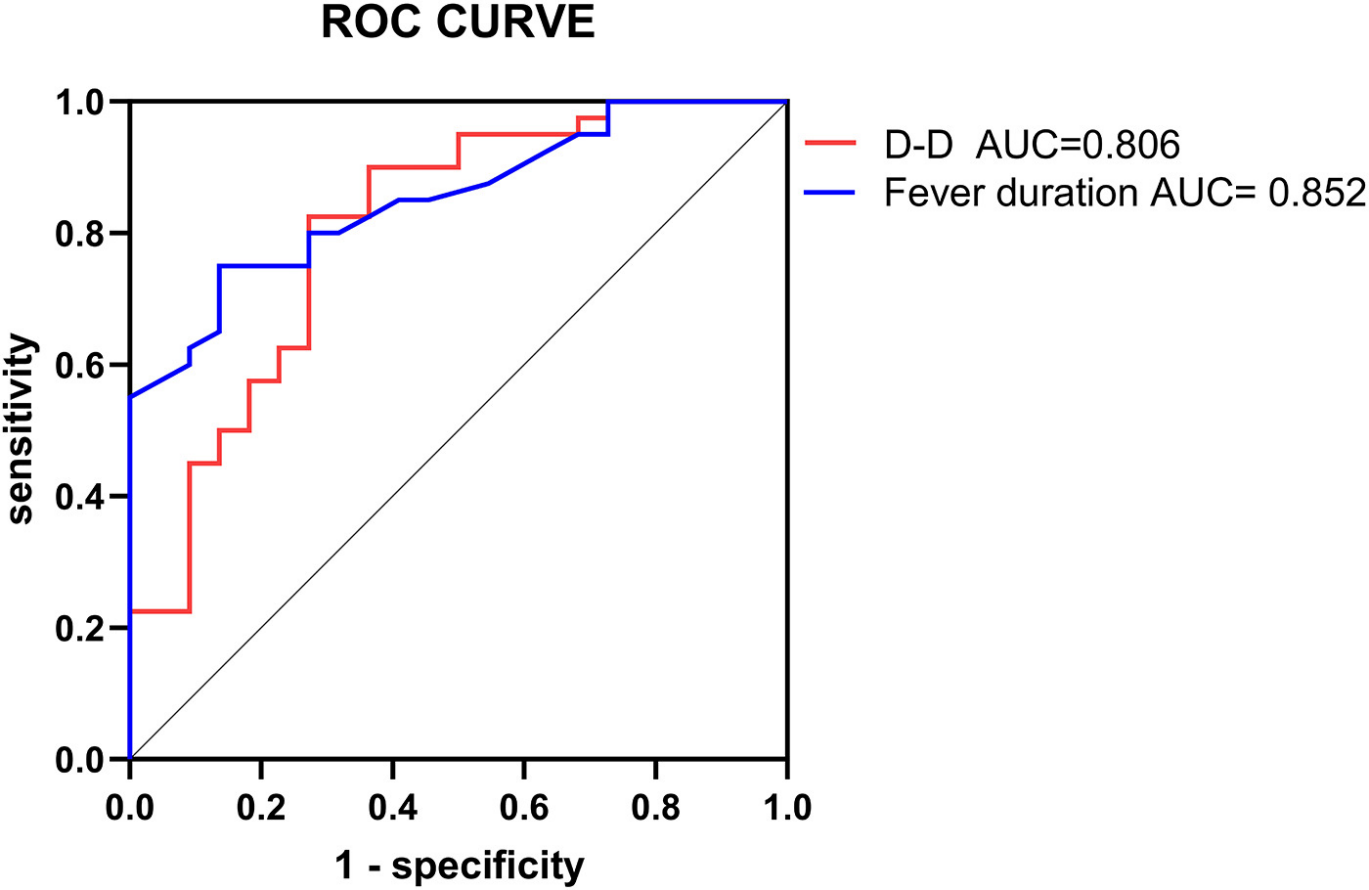
ROC curve of independent risk factors.

The two independent risk factors identified from the logistic regression analysis were used in the ROC curve analysis to assess their predictive value for the prognosis of pediatric patients with PB. The ROC analysis for fever duration indicated an AUC of 0.852, with a cutoff value of 10.55 days and a sensitivity and specificity of 75.0% and 86.4%, respectively. With respect to D-dimer, the ROC analysis revealed an AUC of 0.806, with a cutoff value of 5.61 µg/ml and a sensitivity and specificity of 82.5% and 72.7%, respectively (Table 3, Figure 1).

**Table 3:** ROC curve analysis of independent risk factors.

Factors	AUC	95% CI	Sensitivity	Specificity	Cutoff	*P*
Fever duration	0.852	0.761–0.944	75.0%	86.4%	10.55	<0.001
D-D level	0.806	0.687–0.924	82.5%	72.7%	5.61	<0.001

### 3.6 Changes in pulmonary imaging findings and pulmonary function test results at different stages

**Figure 2:**
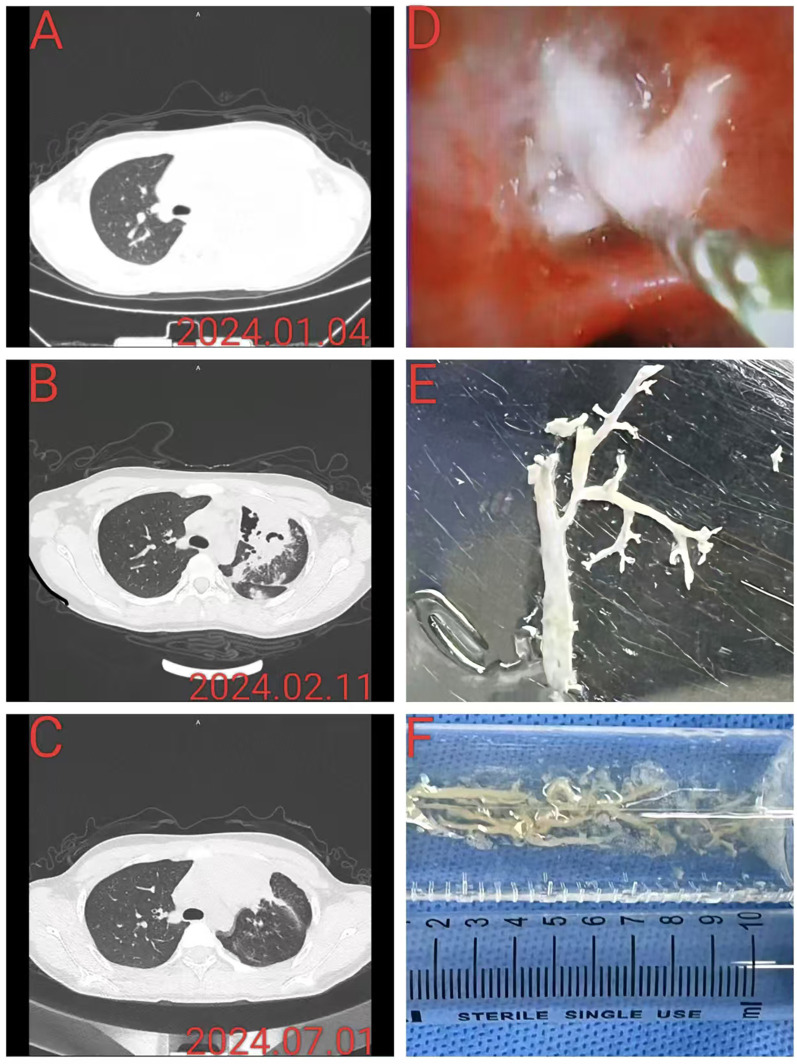
This patient had severe *Mycoplasma pneumoniae* pneumonia complicated by plastic bronchitis. **(A)** A chest CT at admission revealed left upper lobe consolidation. **(B)** Chest CT reexamination approximately 1 month after discharge revealed partial pulmonary necrosis in the left upper lobe, accompanied by bronchopneumonia. **(C)** Chest CT re-examination approximately 6 months after discharge revealed localized atelectasis in the left upper lobe, accompanied by slight chronic inflammation. **(D)** Under bronchoscopy, white rubber-like sputum plug blockage and foreign body forceps removal were performed. **(E,F)** The plastic object with a tree-like shape was removed and placed in a saline tray, and the longest branch was approximately 7 cm long.

The results of pulmonary imaging are as follows: The pulmonary imaging findings were categorized into two types: lesions and resolved. Within 24 h of admission, pulmonary lesions were observed in 100% of the patients, all of whom exhibited bronchopneumonia combined with pulmonary consolidation, in addition to 40 patients with atelectasis (64.5%) and 28 patients with pleural effusion (45.2%). One month after discharge, 33 patients (53.2%) still presented with pulmonary lesions upon reexamination, including 22 patients with localized atelectasis and 19 patients with bronchopneumonia. Among them, 8 patients had localized atelectasis combined with bronchopneumonia, and 1 patient had pneumonia accompanied by pulmonary necrosis. Six months after discharge, 13 patients (21.0%) still had pulmonary lesions upon reexamination, including 6 patients with bronchiectasis, 5 patients with localized atelectasis, and 3 patients with bronchiolitis obliterans. Among them, there was 1 case of localized atelectasis combined with bronchiectasis, and another case of localized atelectasis accompanied by a small amount of chronic inflammation (Table 4 and Figure 2).

**Table 4:** Manifestations of pulmonary dysfunction at different stages according to imaging findings.

Time	Within 24 h of admission (*n* = 62)	One month after discharge (*n* = 62)	Six months after discharge (*n* = 62)
Pulmonary lesions			
Bronchopneumonia combined with pulmonary consolidation (*n*, %)	62 (100%)	0	0
Atelectasis (*n*, %)	40 (64.5%)	0	0
Pleural effusion (*n*, %)	28 (45.2%)	0	0
Pneumonia combined with atelectasis and pleural effusion (*n*, %)	23 (37.1%)	0	0
Localized atelectasis (*n*, %)	0	22 (35.5%)	5 (8.1%)
Bronchopneumonia (*n*, %)	0	19 (30.6%)	0
Pneumonia combined with atelectasis (*n*, %)	0	8 (12.9%)	0
Pneumonia combined with pulmonary necrosis (*n*, %)	0	1 (1.6%)	0
Bronchiolitis obliterans (*n*, %)	0	0	3 (4.8%)
Bronchiectasis (*n*, %)	0	0	6 (9.7%)
Localized atelectasis combined with bronchiectasis (*n*, %)	0	0	1 (1.6%)
Localized atelectasis combined with chronic inflammation (*n*, %)	0	0	1 (1.6%)

**Figure 3:**
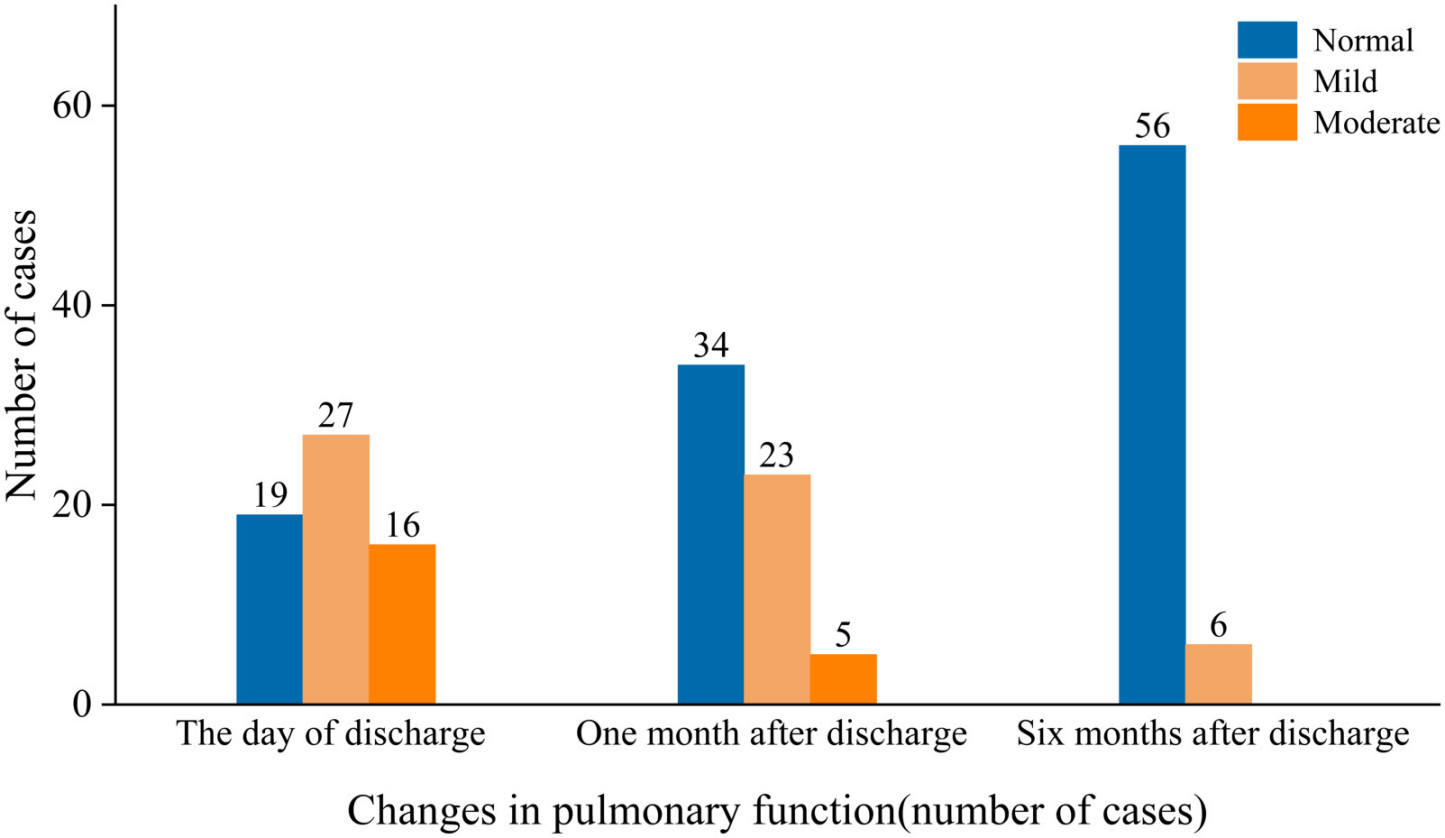
The manifestations of pulmonary function impairment at different stages were classified as normal, mild, or moderate.

The results of pulmonary function testing were as follows: On the day of discharge, 43 patients (69.4%) had abnormal pulmonary function tests, all of whom showed obstructive ventilatory dysfunction, with 27 patients (43.6%) having mild pulmonary dysfunction and 16 patients (25.8%) having moderate pulmonary dysfunction. One month after discharge, 28 patients had abnormal pulmonary function upon reexamination: 23 patients (37.1%) had mildly abnormal pulmonary function, including 16 patients with obstructive ventilatory dysfunction and 7 patients with mixed ventilatory dysfunction; 5 patients (8.1%) had moderately abnormal pulmonary function, all of whom had mixed ventilatory dysfunction. Six months after discharge, 6 patients (9.7%) had abnormal pulmonary function, which was classified as mildly abnormal, manifesting as small airway dysfunction in all 6 patients (Figure 3). The numbers of patients with abnormal pulmonary imaging or pulmonary function at one month and six months after discharge were 40 (64.5%) and 13 (21.0%), respectively.

## 4 Discussion

PB is a rare respiratory disease in children, and its clinical manifestations include fever, cough, and respiratory distress, which can lead to respiratory failure or even death in severe cases (4, 7, 15). The condition is marked by the presence of a jelly like viscous substance that partially or completely obstructs the airways, forming bronchial casts and resulting in impaired pulmonary ventilation (7, 16, 17). These casts, resembling tree branches when placed in saline after bronchoscopic extraction, are responsible for the disease's name (18). The pathological types of PB can be divided into type I and type II (19). Type I is the inflammatory type, mainly showing a large number of inflammatory cell infiltrations and fibrin deposits, with MP, adenovirus and influenza virus all playing important roles (20–22). Type II is the non-inflammatory type, characterized mainly by the accumulation of fibrin and mucin, and is commonly seen in children after congenital heart disease surgery, especially Fontan surgery (23–25).

Although the clinical features of PB are recognized, research on the risk factors for poor prognosis and the medium- to long-term outcomes of PB is relatively scarce. The aim of this study was to fill this gap. By grouping pediatric patients based on their clinical manifestations one month after discharge and conducting a comparative analysis of clinical data between the two groups, we found that a fever duration exceeding 10.55 days and a D-D level above 5.61 µg/ml are independent risk factors for poor prognosis in children with MPP complicated by PB. Additionally, a six-month follow-up of PB patients was conducted to monitor changes in pulmonary imaging and pulmonary function, aiding clinicians in gaining a more comprehensive understanding of the natural course of PB.

Previous studies (4, 5) have indicated that a significant increase in inflammatory markers such as fever duration, CRP, D-D, and LDH in children with MPP is associated with the development of PB caused by MP. MP, a common exogenous pyrogen, stimulates the production of numerous inflammatory factors in the body, including IL-6, IL-10, and IFN-γ (26, 27), potentially leading to persistent fever or even hyperpyrexia. A study by Zhong (5) revealed that the fever duration in children with PB caused by MP (9.55 days) was longer than that in non-PB children (7.79 days); moreover, in a study by Hu (22), the fever duration in PB children due to influenza pneumonia (8 days) exceeded that in non-PB children (6 days). In our study, the average fever duration in PB patients was 11.05 days, which is longer than the fever duration reported in Hu's study for PB patients, suggesting that fever duration may vary with different causative pathogens of PB. However, previous studies have not thoroughly explored the impact of these factors on the prognosis of PB in pediatric patients. The results of our study revealed that PB patients in the poor prognosis group had a fever duration of 11.82 days, which was significantly longer than that in the good prognosis group (9.63 days). Furthermore, the risk of poor prognosis was significantly increased in patients with a fever duration exceeding 10.55 days (OR = 2.871), confirming the association between fever duration and poor prognosis in PB caused by MP.

D-D, a fibrin degradation product reflects the abnormal activation of the body's coagulation and fibrinolytic systems (28, 29). This hypercoagulable state may lead to the formation of microthrombi in the lungs (30, 31), thereby exacerbating the inflammatory response and pulmonary tissue damage in children with PB. A study on the correlation between D-D levels and clinical manifestations in children with MPP revealed that the severely elevated D-D group (D-D > 5.5 µg/ml) had a longer fever duration, more severe pulmonary imaging findings, and a greater likelihood of progressing to severe MPP than did the moderately elevated group (D-D between 0.55 and 5.5 µg/ml) and the normal group (D-D <0.55 µg/ml) (32). Our study revealed that the D-D level in the poor PB prognosis group (6.96 µg/ml) was significantly higher than that in the good prognosis group (4.11 µg/ml), and the higher the D-D level was, the greater the risk of poor prognosis would be (OR = 1.77). These findings suggest that in the clinical setting, for children with PB caused by MP and a fever duration exceeding 10.55 days or D-D levels above 5.61 µg/ml, there should be high suspicion for poor prognosis, and timely bronchoscopic examination and treatment should be conducted, with multiple interventional treatments if necessary. Concurrently, a full course of antibiotic therapy for infection and heparin anticoagulation should be administered.

Through systematic follow-up, the detailed changes in pulmonary imaging and pulmonary function in children with PB from admission to six months postdischarge were identified. At admission, we found that all the children had abnormalities on pulmonary imaging, manifesting mainly as extensive lung consolidation, atelectasis, and pleural effusion, which is consistent with the widespread pulmonary imaging abnormalities reported in previous studies of pediatric PB patients (15, 20, 33). Additionally, on the day of discharge, 69.4% of the enrolled children had pulmonary function tests showing varying degrees of obstructive ventilatory dysfunction, indicating that impaired pulmonary function is relatively common in children with PB caused by MP. Follow-up at one month postdischarge revealed that despite treatment, pulmonary imaging revealed that 53.2% of the children still had abnormalities, and 45.2% had abnormal pulmonary function tests, suggesting that the impact of PB caused by MP on pulmonary structure and pulmonary function may be more prolonged than anticipated, with significant pathological changes potentially persisting in the lungs of children with PB even after the acute symptoms subsided. Further follow-up at six months postdischarge revealed that 21.0% of the children still had pulmonary lesions or impaired pulmonary function; the decrease in this proportion indicates that over time, the pulmonary condition of most children improved. However, some children still had persistent abnormalities at six months, manifesting mainly as localized atelectasis, bronchiolitis obliterans, bronchiectasis, and small airway dysfunction, which is consistent with the results of previous related studies (7, 34, 35). These long-term pulmonary abnormalities may lead to chronic cough, exercise-induced wheezing, pulmonary insufficiency, and other sequelae, affecting the quality of life of children.

Therapeutically, bronchoscopic extraction or removal of casts serves as an essential approach to improve ventilation in pediatric PB patients (36). Anti-infection therapy, corticosteroid-based anti-inflammatory treatment, and mucolytic administration are equally critical. When necessary, repeat bronchoscopic intervention may be performed (7). Furthermore, transvenous retrograde thoracic duct embolization represents a viable therapeutic option for recurrent PB following Fontan palliation (25). Sustained management of PB patients requires ongoing vigilance, with regular follow-up examinations and assessments necessary even after discharge to ensure timely detection and intervention for potential complications.

In conclusion, our study results emphasize the importance of long-term monitoring for children with PB and provide a basis for clinicians to develop personalized intervention strategies to optimize patient long-term management and rehabilitation therapy. Although our study has made certain progress in the prognostic analysis of pediatric PB caused by MP, the single-center design and limited sample size may limit the robustness of the results. Moreover, the single-pathogen cohort constrains the generalizability of findings to PB cases caused by other infectious agents. Therefore, we suggest that future studies with a wider etiological spectrum, a broader sample size and in a multicenter context be conducted to address the limitations of our findings. Moreover, since the follow-up period spanned only one month and six months after discharge, we were unable to assess the longer-term prognosis of PB fully. Consequently, extending the duration of follow-up is essential to uncover the long-term effects of PB. Despite these limitations, our study is the first to provide a detailed account of pulmonary function in children with PB caused by MP postdischarge, enriching the clinical phenotypic spectrum of pediatric PB and offering a scientific basis for long-term management.

## 5 Conclusions

Our study revealed that a fever duration exceeding 10.55 days and a D-dimer level exceeding 5.61 µg/ml are independent risk factors for poor prognosis in children with MPP complicated by PB. These findings are important for the early identification of pediatric patients at risk of poor prognosis and the implementation of timely and effective treatment measures. Given the potential for long-term pulmonary function abnormalities and sequelae in children with PB caused by MP, we underscore the need for intensified follow-up and the formulation of personalized intervention strategies.

## Data Availability

The raw data supporting the conclusions of this article will be made available by the authors, without undue reservation.
